# Supplementary material for: A gene regulatory network controls the balance between mesendoderm and ectoderm at pluripotency exit
Source: Mol Syst Biol. 2019 Dec 6;15(12):e9043. doi: 10.15252/msb.20199043 (PMC6896232; doi:10.15252/msb.20199043)
Supplement: Supplementary file 1 — Appendix [file MSB-15-e9043-s001.pdf]

Appendix to *A gene regulatory network controls the balance between mesendoderm and ectoderm at pluripotency exit*

Hanna L Sladitschek, Pierre A Neveu

Contents

Appendix Figure S1 2

Appendix Figure S2 3

Appendix Figure S3 4

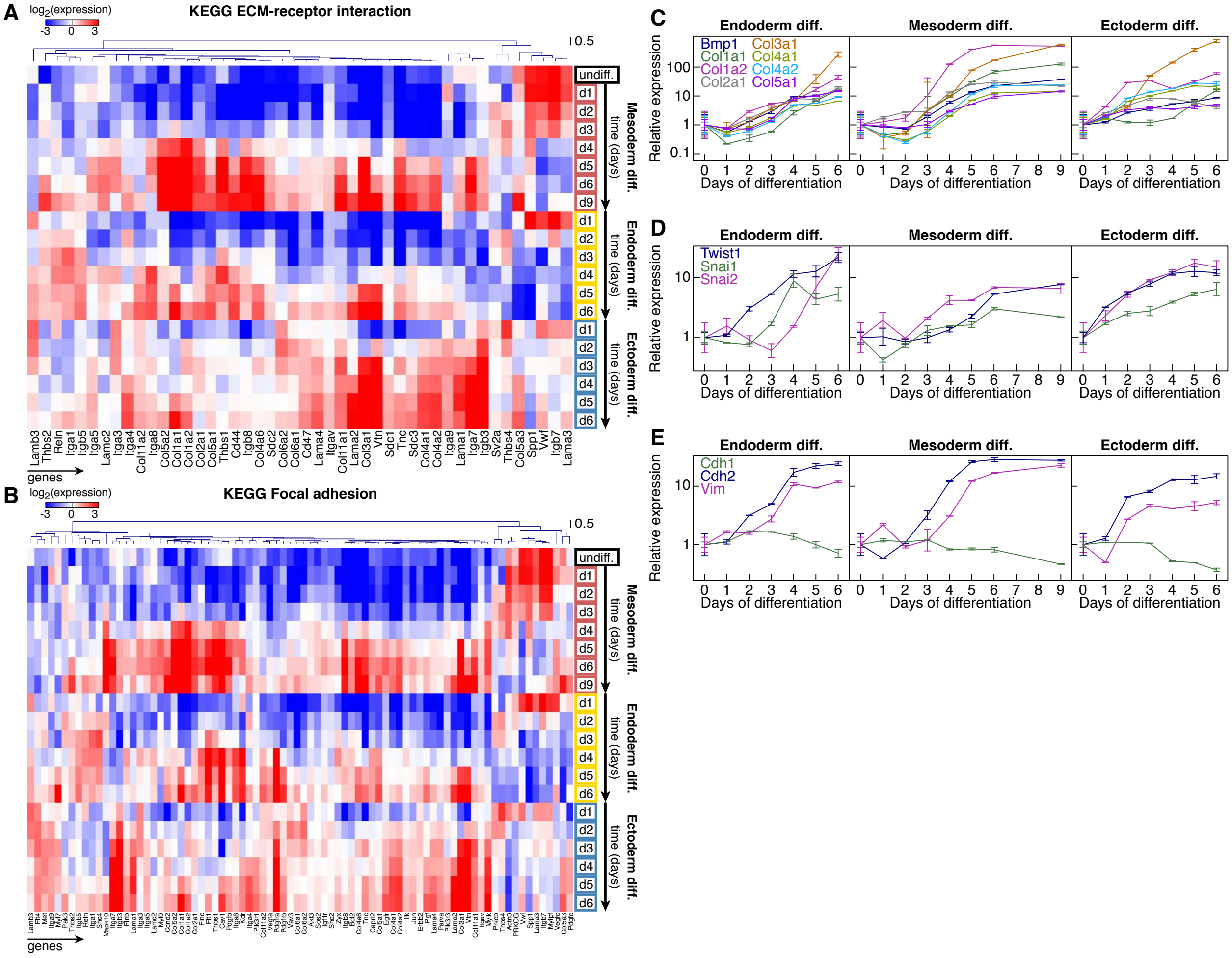

Appendix Figure S1. Expression of genes associated with the extracellular matrix, cell adhesion and epithelial-mesenchymal transition.

A. Hierarchical clustering of mRNA expression of genes belonging to the KEGG pathway "Extracellular matrix-receptor interactions" differentially expressed during the differentiation of mESCs towards the three germ layers.

B. Hierarchical clustering of mRNA expression of genes belonging to the KEGG pathway "Focal adhesion" differentially expressed during the differentiation of mESCs towards the three germ layers.

C. Expression time course of collagen genes and BMP1 during differentiation towards the three germ layers (n= 2. Data represented as mean $\pm$ S.D.).

D. Expression time course of Twist (Twist1), Snail (Snai1) and Slug (Snai2) during differentiation towards the three germ layers (n= 2. Data represented as mean $\pm$ S.D.).

E. Expression time course of E-cadherin (Cdh1), N-cadherin (Cdh2) and Vimentin (Vim) during differentiation towards the three germ layers (n= 2. Data represented as mean $\pm$ S.D.).

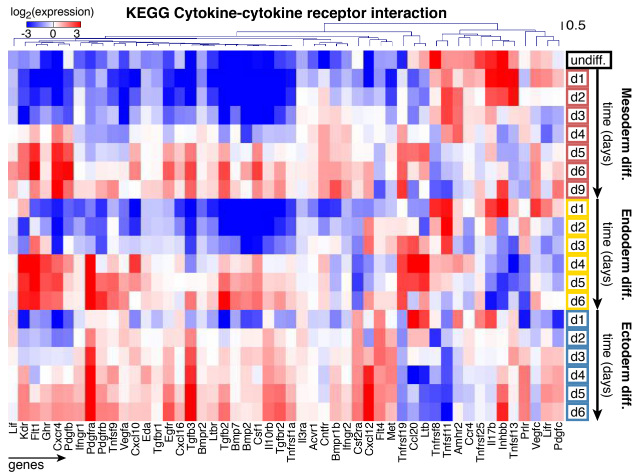

Appendix Figure S2. Expression of genes associated with extracellular signaling.
Hierarchical clustering of mRNA expression of genes belonging to the KEGG pathway “Cytokine-cytokine receptor interactions” differentially expressed during the differentiation of mESCs towards the three germ layers.

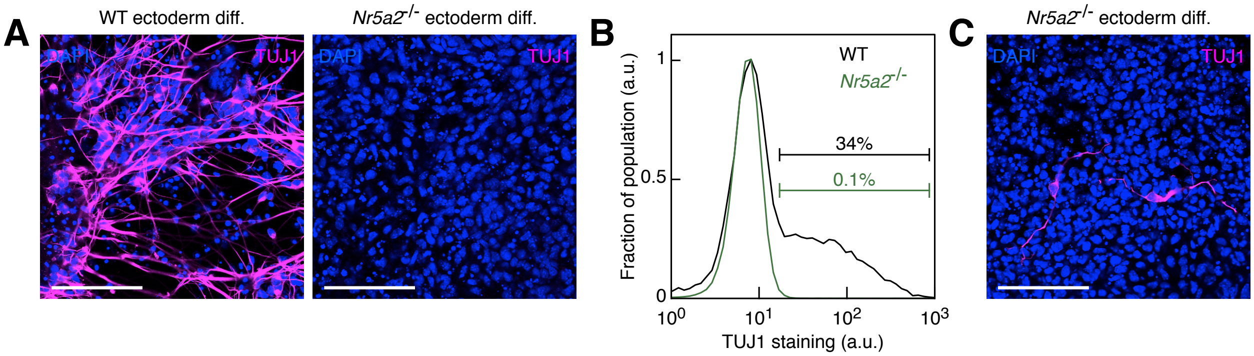

Appendix Figure S3. Ectoderm differentiation is impaired in *Nr5a2*$^{-/-}$ mESCs.

A. Representative TUJ1 immunostaining after six days of differentiation towards ectoderm of wild type mESCs or *Nr5a2*$^{-/-}$ mESCs. Bar: 100 μm.

B. Quantification of TUJ1-positive cells after six days of differentiation towards ectoderm of wild type mESCs (black) or *Nr5a2*$^{-/-}$ mESCs (green) as measured by flow cytometry.

C. Example of TUJ1-positive cells after six days of differentiation towards ectoderm of *Nr5a2*$^{-/-}$ mESCs. Bar: 100 μm.
