# Supplementary material for: A gene regulatory network controls the balance between mesendoderm and ectoderm at pluripotency exit
Source: Mol Syst Biol. 2019 Dec 6;15(12):e9043. doi: 10.15252/msb.20199043 (PMC6896232; doi:10.15252/msb.20199043)
Supplement: Supplementary file 2 — Expanded View Figures PDF [file MSB-15-e9043-s002.pdf]

Expanded View Figures

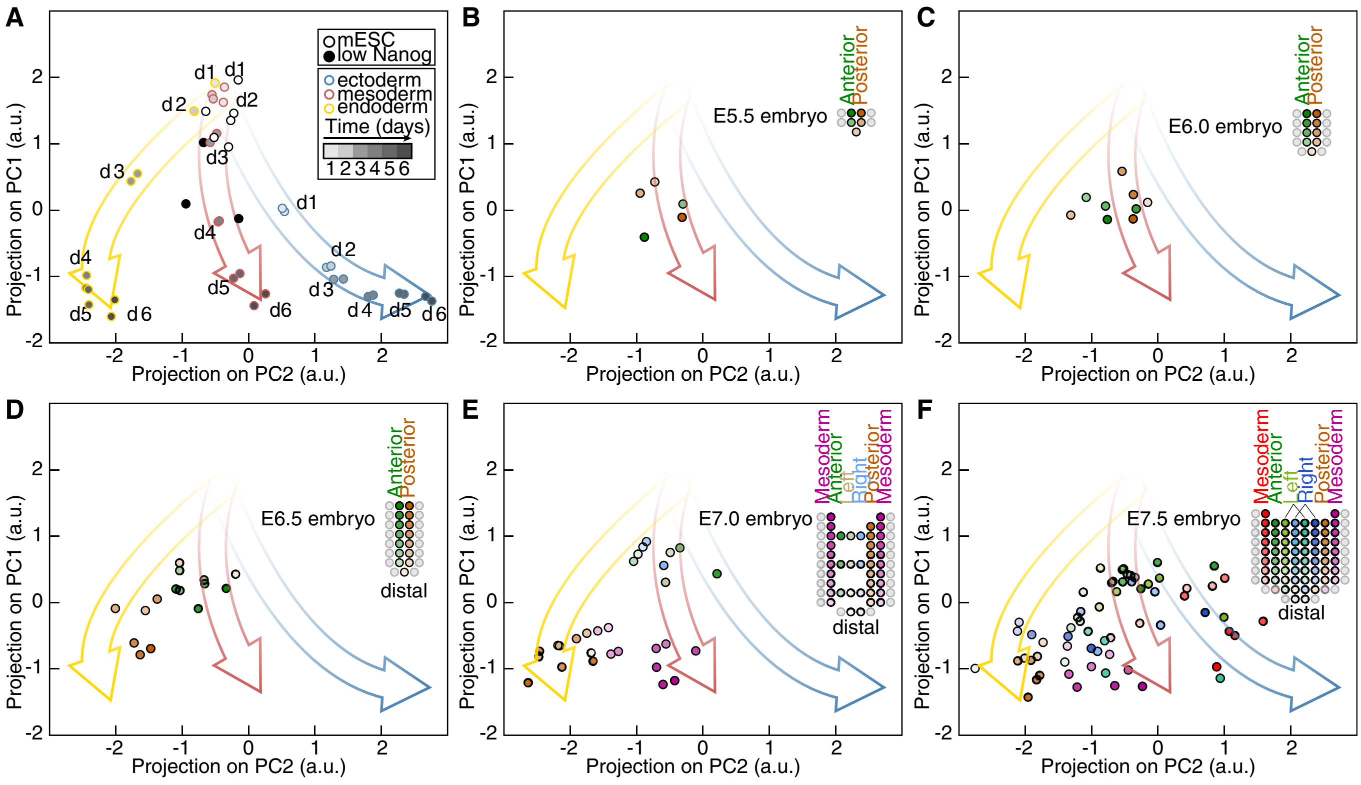

Figure EV1. Comparison of *in vitro* mESC differentiation with transcriptomes originating from spatially defined regions of gastrulating mouse embryos published in Peng *et al* (2019).

A Projection on PC1 and PC2 of gene expression profiles during mESC differentiation to endoderm, mesoderm, and ectoderm and of mESCs with low Nanog expression levels.

B–F Projection on PC1 and PC2 of transcriptomes of spatially defined regions of mouse embryos at E5.5 (B), E6.0 (C), E6.5 (D), E7.0 (E), and E7.5 (F) stages (data from Peng *et al*, 2019). Corresponding embryo regions are indicated according to the color codes in the inset. Sections belonging to the visceral endoderm were not considered.

 © 2019 The Authors

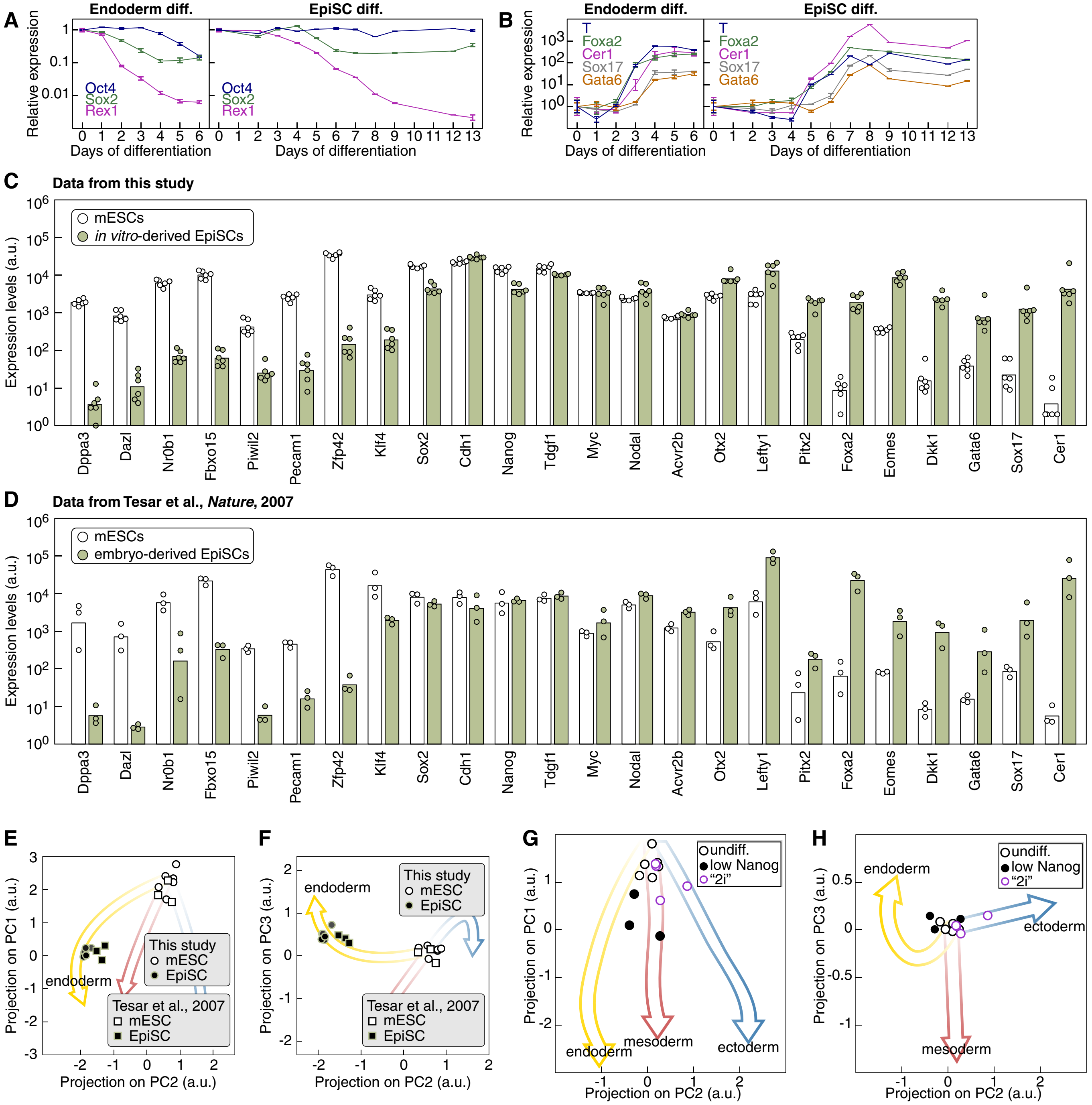

Figure EV2. Expression of marker genes during mESC differentiation to endoderm and to EpiSCs.

A, B Expression time course of pluripotency (A) and endoderm (B) markers during differentiation toward endoderm or EpiSCs ($n = 2$. Data represented as mean ± SD).

C, D Expression levels of naïve and primed pluripotency markers in mESCs and *in vitro*-differentiated EpiSCs (C) or in mESCS and embryo-derived EpiSCs (D, using expression data from Tesar *et al* (2007)).

E, F Projection on PC1 and PC2 (E) or on PC2 and PC3 (F) of gene expression profiles of mESCs, *in vitro*-differentiated EpiSCs from this study (circles) and mESCs and embryo-derived EpiSCs [squares, using expression data from Tesar *et al* (2007)].

G, H Projection on PC1 and PC2 (G) or on PC2 and PC3 (H) of gene expression profiles of mESCs with low Nanog expression levels (black) or mESCs maintained in "2i" (purple).

© 2019 The Authors

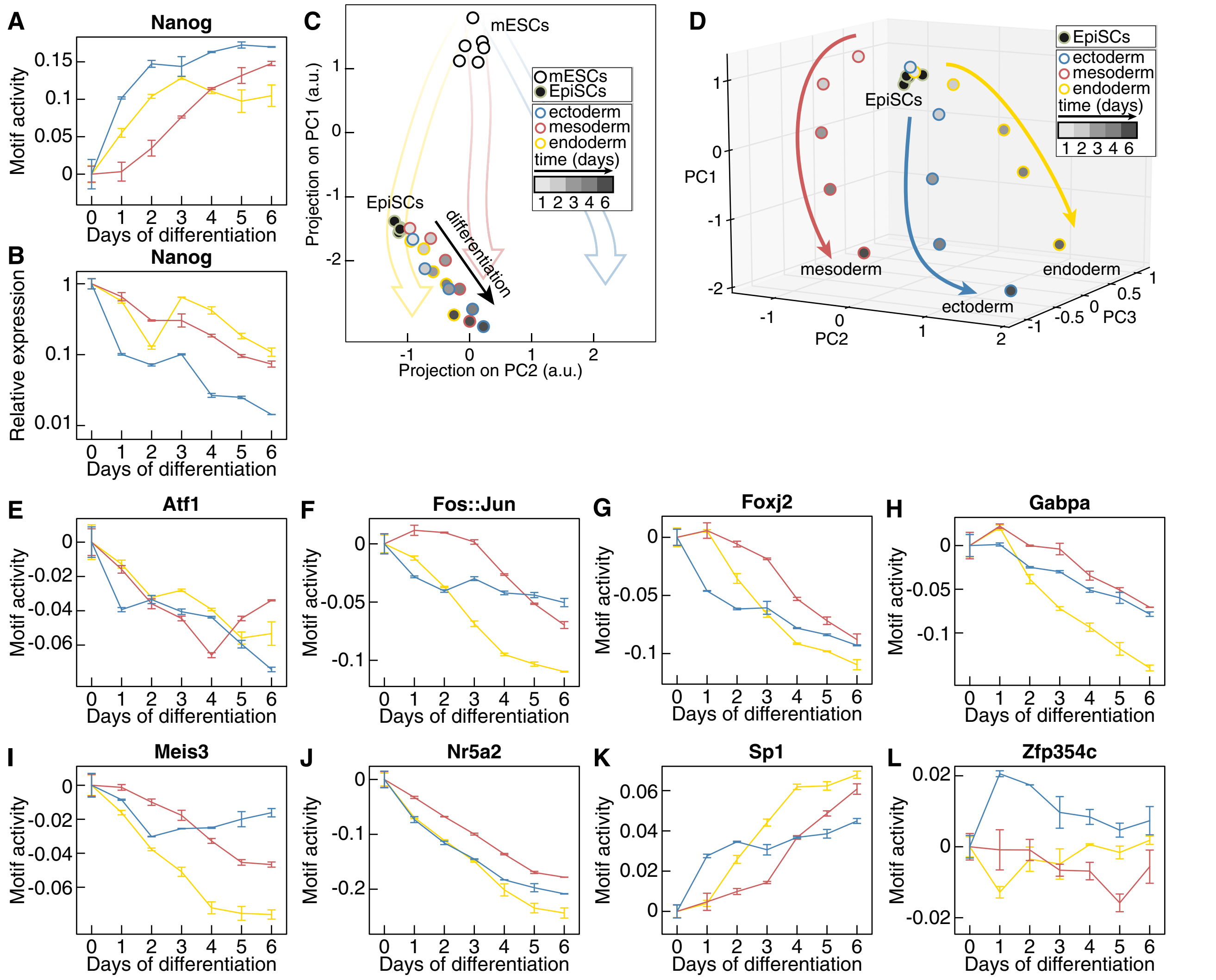

Figure EV3. Comparison of motif activity and gene expression trajectories during mESC differentiation to the three germ layers.

A, B Motif activity (A) and mRNA expression levels (B) of Nanog during mESC differentiation to the three germ layers (yellow: endoderm differentiation, red: mesoderm differentiation, blue: ectoderm differentiation. $n = 2$. Data represented as mean ± SD).

C Projection on PC1 and PC2 (computed using mESC differentiation data) of gene expression profiles during EpiSC differentiation.

D PCA of gene expression changes during EpiSC differentiation.

E–L Motif activity of the highly connected nodes Atf1 (E), Fos::Jun (F), Foxj2 (G), Gabpa (H), Meis3 (I), Nr5a2 (J), Sp1 (K), and Zfp354c (L) during mESC differentiation to the three germ layers (yellow: endoderm differentiation, red: mesoderm differentiation, blue: ectoderm differentiation. $n = 2$. Data represented as mean ± SD).

 © 2019 The Authors

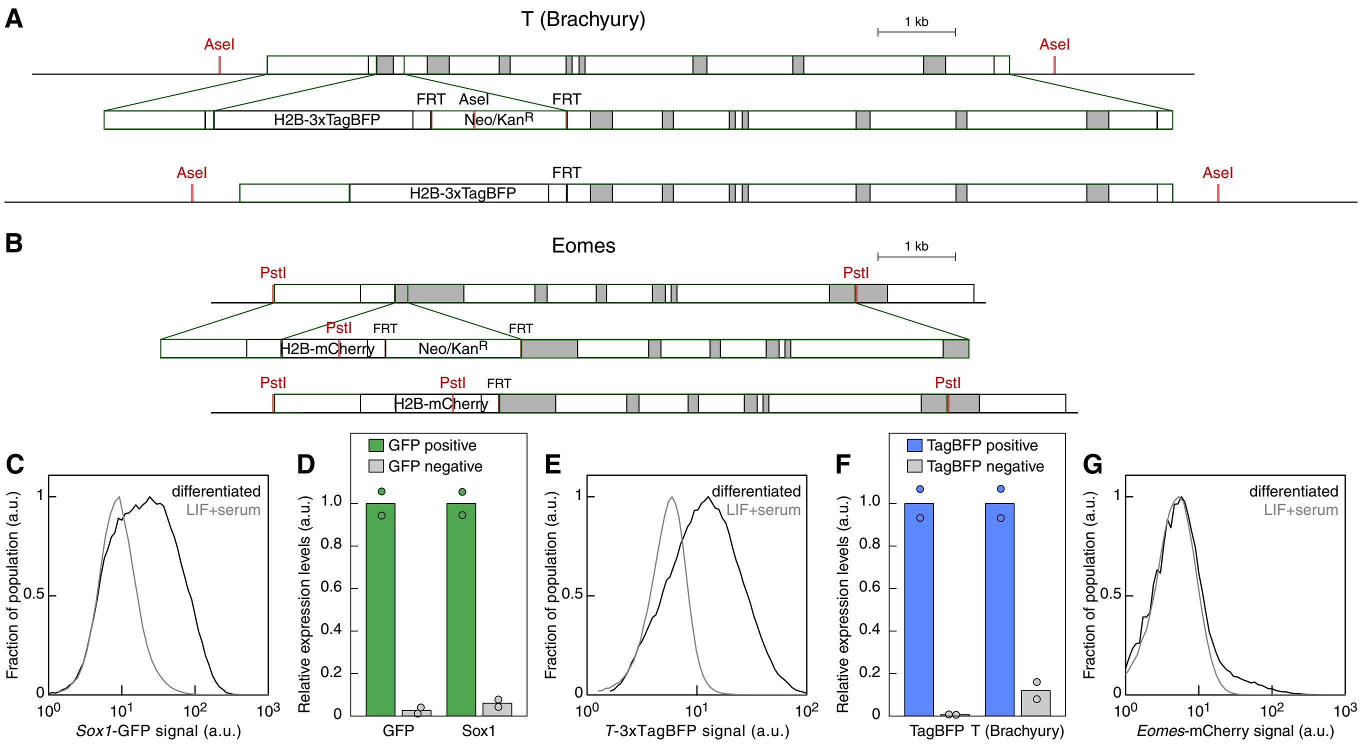

Figure EV4. Generation of a triple knock-in *Sox1-Brachyury-Eomes* reporter mESC line.

- A Scheme of the T (also known as Brachyury) targeting construct and resulting genomic locus after integration by homologous recombination and subsequent removal of the selection cassette by Flippase expression.
- B Scheme of the Eomes targeting construct and resulting genomic locus after integration by homologous recombination and subsequent removal of the selection cassette by Flippase expression.
- C *Sox1*-GFP signal as measured by flow cytometry in undifferentiated 3KI mESCs (gray, "LIF+serum") or differentiated to ectoderm (black).
- D GFP and Sox1 expression levels as measured by mRNA-Seq in FACS-purified GFP-positive or GFP-negative cells. Expression levels were normalized to the mean expression in differentiating FACS-purified positive cells for each gene.
- E *T*-TagBFP signal as measured by flow cytometry in undifferentiated 3KI mESCs (gray, "LIF+serum") or differentiated to mesendoderm (black).
- F TagBFP and T (Brachyury) expression levels as measured by mRNA-Seq in differentiating FACS-purified TagBFP-positive or TagBFP-negative cells. Expression levels were normalized to the mean expression in FACS-purified positive cells for each gene.
- G *Eomes*-mCherry signal as measured by flow cytometry in undifferentiated 3KI mESCs (gray, "LIF+serum") or differentiated to definitive endoderm (black).

Figure EV5. Generation of *Atf1*$^{-/-}$, *Fos*$^{-/-}$*Jun*$^{-/-}$, *Foxj2*$^{-/-}$, *Meis3*$^{-/-}$, *Nr5a2*$^{-/-}$, *Sp1*$^{+/-}$, and *Zfp354c*$^{-/-}$ mESCs.

- A Genomic locus of *Atf1* (exons are indicated in gray, the position of the guide RNA—gRNA—in red), Sanger sequencing result of the alleles obtained by CRISPR/Cas9 and Western blot analysis of the parental cell line and *Atf1*$^{-/-}$ line (marker sizes in kDa are indicated, the anti-ATF1 antibody detects both ATF1 and CREB-1).
- B Genomic locus of *Fos* and *Jun* (exons are indicated in gray, the position of the guide RNA—gRNA—in red) and Sanger sequencing result of the alleles obtained by CRISPR/Cas9 and Western blot analysis of the parental cell line and *Fos*$^{-/-}$*Jun*$^{-/-}$ line (marker sizes in kDa are indicated).
- C Genomic locus of *Foxj2* (exons are indicated in gray, the position of the guide RNA—gRNA—in red) and Sanger sequencing result of the alleles obtained by CRISPR/Cas9 and Western blot analysis of the parental cell line and *Foxj2*$^{-/-}$ line (marker sizes in kDa are indicated).
- D Genomic locus of *Meis3* (exons are indicated in gray, the position of the guide RNA—gRNA—in red) and Sanger sequencing result of the alleles obtained by CRISPR/Cas9 and Western blot analysis of the parental cell line and *Meis3*$^{-/-}$ line.
- E Genomic locus of *Nr5a2* (exons are indicated in gray, the position of the guide RNA—gRNA—in red) and Sanger sequencing result of the alleles obtained by CRISPR/Cas9 and Western blot analysis of the parental cell line and *Nr5a2*$^{-/-}$ line (marker sizes in kDa are indicated).
- F Genomic locus of *Sp1* (exons are indicated in gray, the position of the guide RNA—gRNA—in red) and Sanger sequencing result of the alleles obtained by CRISPR/Cas9 and Western blot analysis of the parental cell line and *Sp1*$^{+/-}$ line (marker sizes in kDa are indicated).
- G Genomic locus of *Zfp354c* (exons are indicated in gray, the position of the guide RNA—gRNA—in red) and Sanger sequencing result of the alleles obtained by CRISPR/Cas9 and Western blot analysis of the parental cell line and *Zfp354c*$^{-/-}$ line (marker sizes in kDa are indicated).

© 2019 The Authors

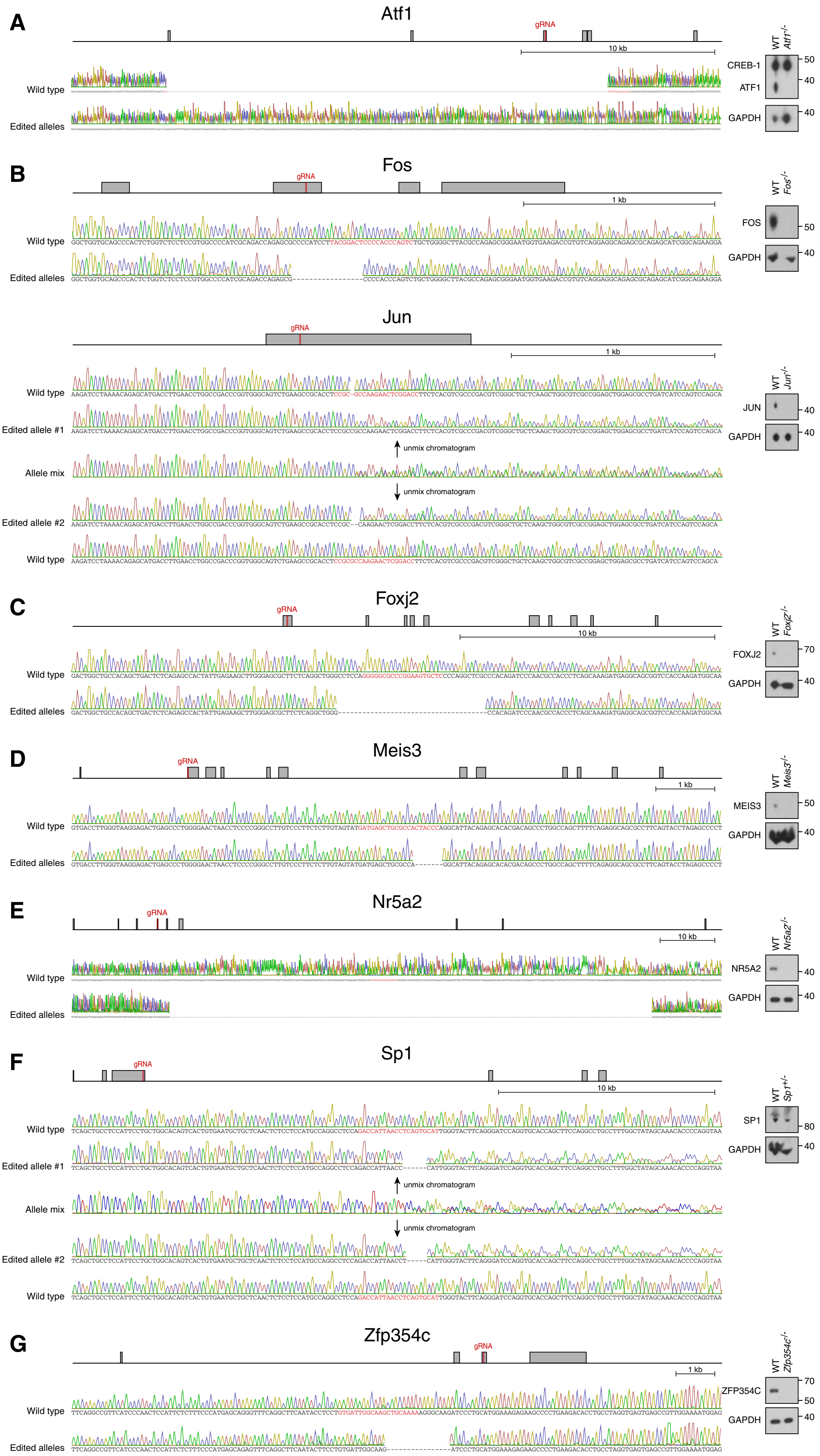

Figure EV5.

 © 2019 The Authors
